# Gram-Scale Domino Synthesis in Batch and Flow Mode of Azetidinium Salts

**DOI:** 10.1021/acs.joc.1c01487

**Published:** 2021-09-01

**Authors:** Alessandra Sivo, Vincenzo Ruta, Gianvito Vilé

**Affiliations:** Department of Chemistry, Materials, and Chemical Engineering “Giulio Natta”, Politecnico di Milano, Piazza Leonardo da Vinci 32, IT-20133 Milano, Italy

## Abstract

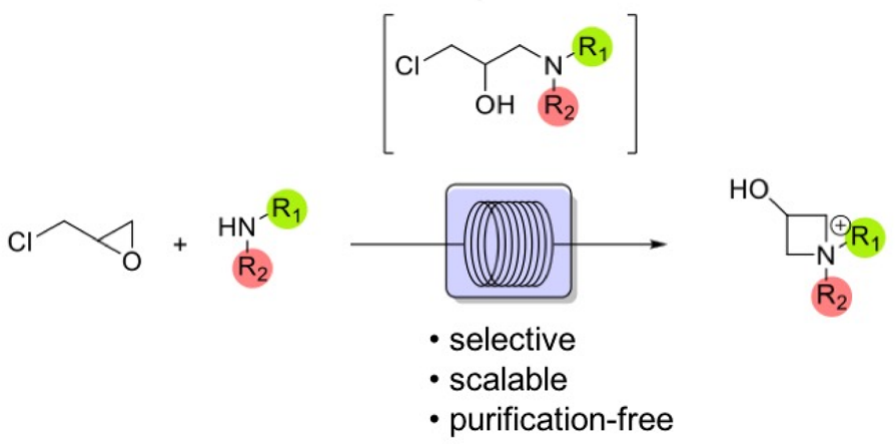

Azetidinium salts are important motifs in organic synthesis but are difficult to obtain due to extremely long synthetic protocols. Herein, a rapid continuous-flow process for the on-demand synthesis of azetidinium salts is described. In particular, the nucleophilic addition of secondary amines and the subsequent intramolecular N-cyclization have been investigated in batch and continuous-flow modes, exploring the effects of solvent type, temperature, reaction time, and amine substituent on the synthesis of azetidinium salts. This has enabled us to quickly identify optimal reaction conditions and obtain microkinetic parameters, verifying that the use of a flow reactor leads to a reduction of the activation energy for the epichlorohydrin aminolysis due to the better control of mass and heat transfer during reaction. This confirms the key role of continuous-flow technologies to affect the kinetics of a reaction and make synthetic protocols ultrarapid and more efficient.

## 1 Introduction

**Scheme 1:**
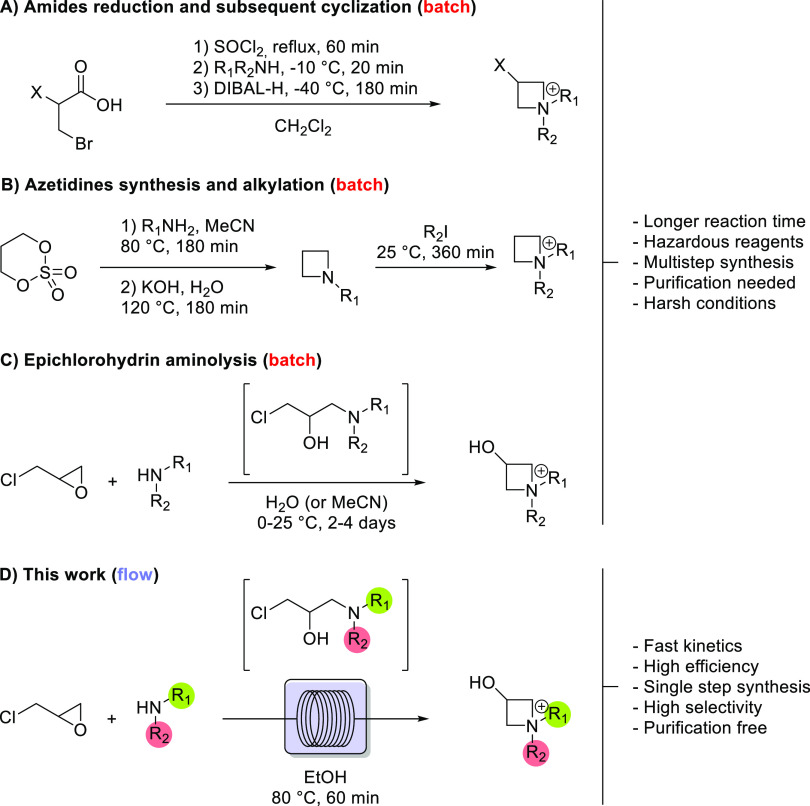
General Strategies for Azetidinium Synthesis

Fragment-based drug design is becoming a paradigm for pharmaceutical synthesis.^1^ However, the lack of molecular rigidity intrinsic to a majority of small molecules appears to be critical for the implementation of this approach. One of the most popular ways to limit molecular conformational flexibility relies on the introduction of saturated three- or four-membered nitrogen heterocycles. In this context, azetidinium salts are widely known for their versatility and peculiar reactivity: because of the ring strain, they are commonly used for alkylation via ring opening reactions with C, N, S, and O nucleophiles, and are also employed in the generation of substituted pyrrolidines via ring expansion reaction.^2−4^ The usefulness of azetidinium salts has been proven in several fields, from pharmaceutical synthesis to polymer branching.^5^ In particular, they have been employed in the synthesis of 3-aryloxy-3-aryl-1-propanamines, key intermediates to make selective serotonin reuptake inhibitors such as fluoxetine.^6^ Several studies have examined the development of synthetic protocols for the production of azetidiniums, following principally three pathways: the first via amide formation and subsequent reduction forming an amine that undergoes a ring-closing rearrangement (Scheme 1A),^7^ the second via a ring-closure reaction to give the azetidine, which is then treated with methylating agents (MeOTf,^5^ LiHDMS, or CH_3_I) to give the corresponding azetidinium salt (Scheme 1B),^8^ and the last via epoxide aminolysis using secondary amines (Scheme 1C).^2^ In particular, epoxide aminolysis has been extensively studied and various conditions have been developed, including those based on microwave- and ultrasound-assisted methods, on the use of lanthanide or aluminum triflates and Lewis acids reagents, and on solid acid supports.^9−15^ Among epoxides, epichlorohydrin presents a chlorine atom as a substituent of the epoxy motif and is an essential building block for pharmaceutical and fine chemical synthesis, due to its peculiar reactivity toward nucleophiles. The possibility of easily obtaining from it β-aminoalcohols as key intermediates for β-blockers, like atenolol and propranolol, is one of the most attractive applications of this compound, which today is used also in polymer synthesis to prepare cellulose-based materials.^16−18^

The principal issues for all of these synthetical pathways are the need for harsh conditions (cryogenic and/or reflux), hazardous reagents as starting materials (i.e., SOCl_2_ or DIBAL-H), long and expensive purifications, and potential exothermic steps. In this direction, it is well established that continuous-flow chemistry, in which common batch reactors are replaced by microreactors with tailored geometry, materials, and dimensions, enables better control of the reaction parameters and driving forces, such as temperature, pressure, heat, and mass transfer.^19−36^ In the case of the highly exothermic epichlorohydrin aminolysis, the better control of temperature operated by microreactors allows one to carry out the reaction in a safer way, with important consequences for the reaction yield (Scheme 1D). Finally, the use of a flow reactor meets the need for a safer, environmentally sustainable, and circular chemistry.^37^

By exploiting this technology, we have developed a flow route to synthesize azetidinium salts, elucidating the kinetics of the reaction and optimizing the process to understand the reaction mechanism and the effect of solvation and temperature. We have also compared batch and flow data under optimized conditions to study the effect of the reactor geometry on the kinetics of the process. Finally, we have tested the flexibility of the protocol using different amines, from primary to secondary, evaluating the generality of the method with reactants having steric and electronic substitutions. Overall, the route presented herein is facile and rapid, and enables the synthesis of azetidiniums in gram quantities, making it possible to further explore the chemical space around them for pharmaceutical and fine chemical applications.

## 2 Results and Discussion

### 2.1 Batch Optimization

We have initiated the work by conducting preliminary tests in batch mode, carrying out the aminolysis of epichlorohydrin using diethylamine, at room temperature, for 48 h in water. After dropwise addition of epichlorohydrin **1** to a stirred solution of diethylamine, the obtained 1-chloro-*N*,*N*-(diethylamino)propan-2-ol intermediate **2** spontaneously undergoes an intramolecular cyclization via bimolecular nucleophilic substitution (SN_2_) at C1, giving the corresponding *N*,*N*-(diethyl)-3-hydroxyazetidinium salt **3** in 75% yield. The formation and subsequent disappearance of the intermediate have been elucidated by ^1^H NMR analysis, through the determination of the intensity of the characteristic peaks assigned to this product (dd, δ 3.64–3.70). This approach was the only one that could monitor the reaction, because the degree of conversion of the two reactants could not be calculated given that both epichlorohydrin and diethylamine are volatile species and evaporate during concentration of the samples.

The effects of the solvent (acetonitrile, hexane, ethanol, and water) and temperature (25, 60, and 80 °C in H_2_O) are listed in Table 1. In particular, the yield of **3** increases with temperature from 2% at 25 °C to 51% at 80 °C in EtOH. The effect demonstrates that higher temperatures are needed to activate the nucleophilic addition and the subsequent intramolecular N-cyclization. The solvent can also make a major contribution to the reactivity. In fact, at 60 °C and 60 min, the reaction in EtOH gives a 30% yield and the reaction in H_2_O gives an 71% yield. However, at 60 °C and 60 min, the reaction in acetonitrile gives a 11% yield and the reaction in hexane gives an 19% yield. These results confirm that, differently from the classical solvent effect on SN_2_ reactions, polar protic solvents are the most appropriate choice for the synthesis of azetidinium salts. This can be justified considering the increased level of activation of the epichlorohydrin and stabilization of the obtained aminolysis intermediate, involving pseudocyclic structures that are more stable in polar solvents.^38^ Finally, longer reaction times are also beneficial for the reaction. On the basis of these results, it was possible to identify H_2_O and EtOH as the best solvents and high temperature as a driving force for the reaction, which provided **3** in 83% and 51% overall yields, respectively, at 80 °C in 60 min. Both solvents have peculiar advantages. If water enhances the reaction yield, ethanol provides a better solubilization of the reactants, effectively improving mass transfer and being easier to remove from the reaction mixture.

**Table 1:**
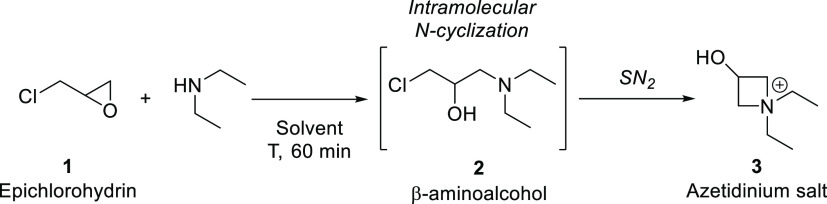
Solvent and Temperature Screening for Azetidinium Synthesis under Batch Conditions^a^

entry	solvent	*T* (°C)	*t* (min)	yield^b^ (%)
1	acetonitrile	60	60	11
2	hexane	60	60	19
3	EtOH	25	60	2
4	EtOH	60	60	30
5	EtOH	80	60	51
6	H_2_O	25	5	3
7	H_2_O	25	30	7
8	H_2_O	25	60	11
9	H_2_O	60	5	42
10	H_2_O	60	30	55
11	H_2_O	60	60	71
12	H_2_O	80	5	69
13	H_2_O	80	30	78
14	H_2_O	80	60	84

a Conditions: 28 mmol of epichlorohydrin, 28 mmol of diethylamine, and 5 mL of solvent.

b Determined by NMR, using dibromomethane as the internal standard.

### 2.2 Development of a Continuous-Flow Process

**Figure 1:**
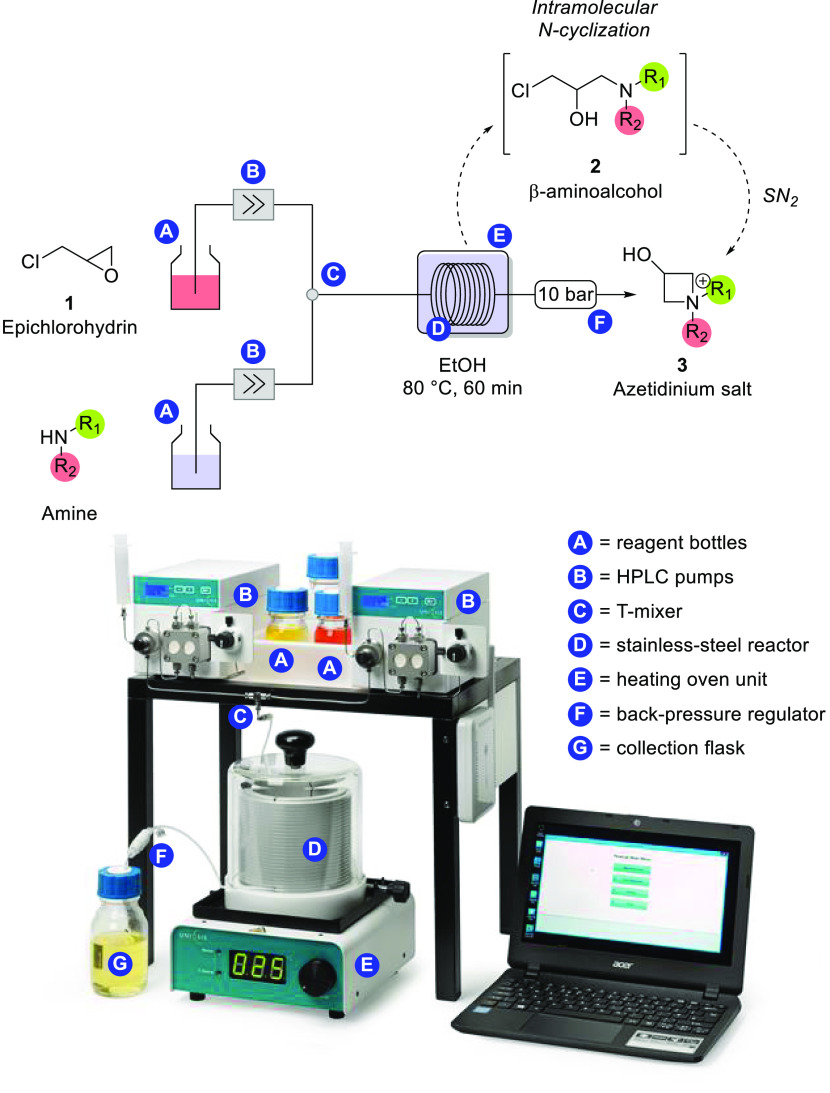
Flow setup for azetidinium synthesis under continuous-flow conditions.

After this preliminary batch optimization, we have considered developing a flow route for the reaction. The choice of a flow reactor is justified on the basis of two main aspects: (i) safety concerns due to the reaction exothermicity (in the batch protocol, epichlorohydrin had to be added dropwise at 0 °C), addressed by the better containment upon operating microreactors as closed systems, and (ii) a more appropriate control of the reaction parameters, particularly heat transfer effects, due to the reactor geometry (Figure 1). For this reason, we have used a commercial UNIQSIS continuous-flow automated platform featuring two HPLC pumps that inject reagent solutions into the flow setup. After passing a T-mixer, reagents flow in a stainless steel coil reactor (with an internal volume of 60 mL) under strictly controlled temperature and pressure conditions regulated by a heating module and a back-pressure regulator valve (10 bar), respectively. This intrinsic automation of the setup simplifies the synthetic protocol, avoiding the tedious and potentially harmful dropwise addition of epichlorohydrin to the amine-stirred solution.

**Figure 2:**
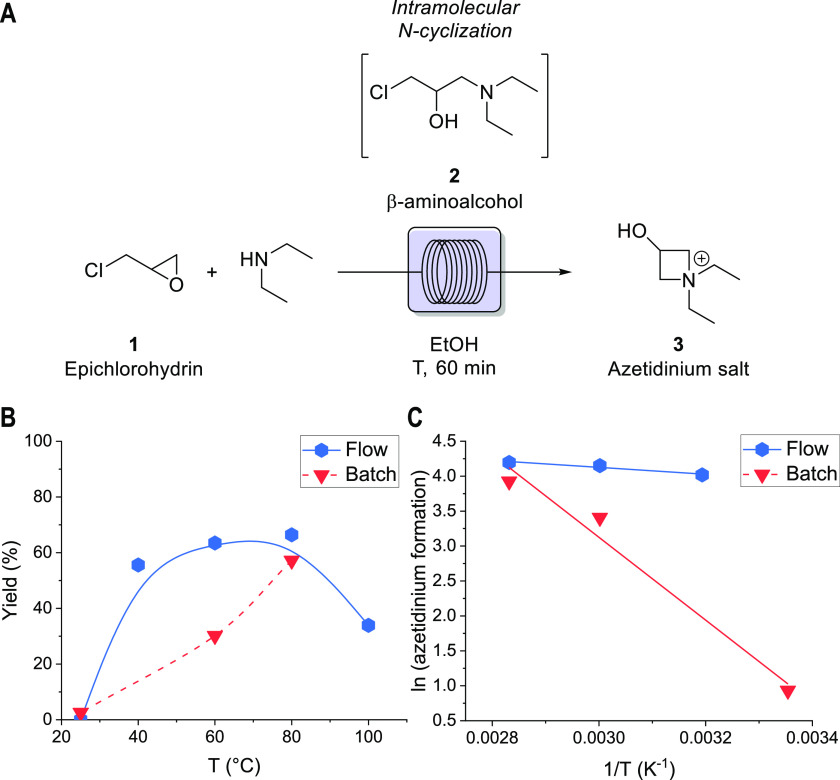
Temperature screening under continuous-flow conditions and comparison with batch data.

To investigate the reaction performance under flow conditions, we have decided to study the effect of temperature on the synthesis of **3**, evaluating the reaction in water and EtOH as solvents, and keeping 60 min as the residence time (Figure 2A). Preliminary tests conducted in water led in all cases to the clogging of the reactor, mainly because of the low solubility of epichlorohydrin in water. To avoid these issues, EtOH was chosen as a solvent. This has enabled us to obtain comparative data between the batch and flow process, showing the increased yields under flow conditions (Figure 2B). The enhancement of the reaction rate is corroborated by the vis-à-vis comparison of batch and flow data in Table S1 and by the values of the activation energy determined by the Arrhenius analysis under kinetic conditions. In fact, the reaction under batch conditions requires an activation energy of 49 kJ mol^–1^, while the same reaction under flow mode needs only 4 kJ mol^–1^ (Figure 2C).

### 2.3 Substrate Scope and Scale-up Analysis

**Figure 3:**
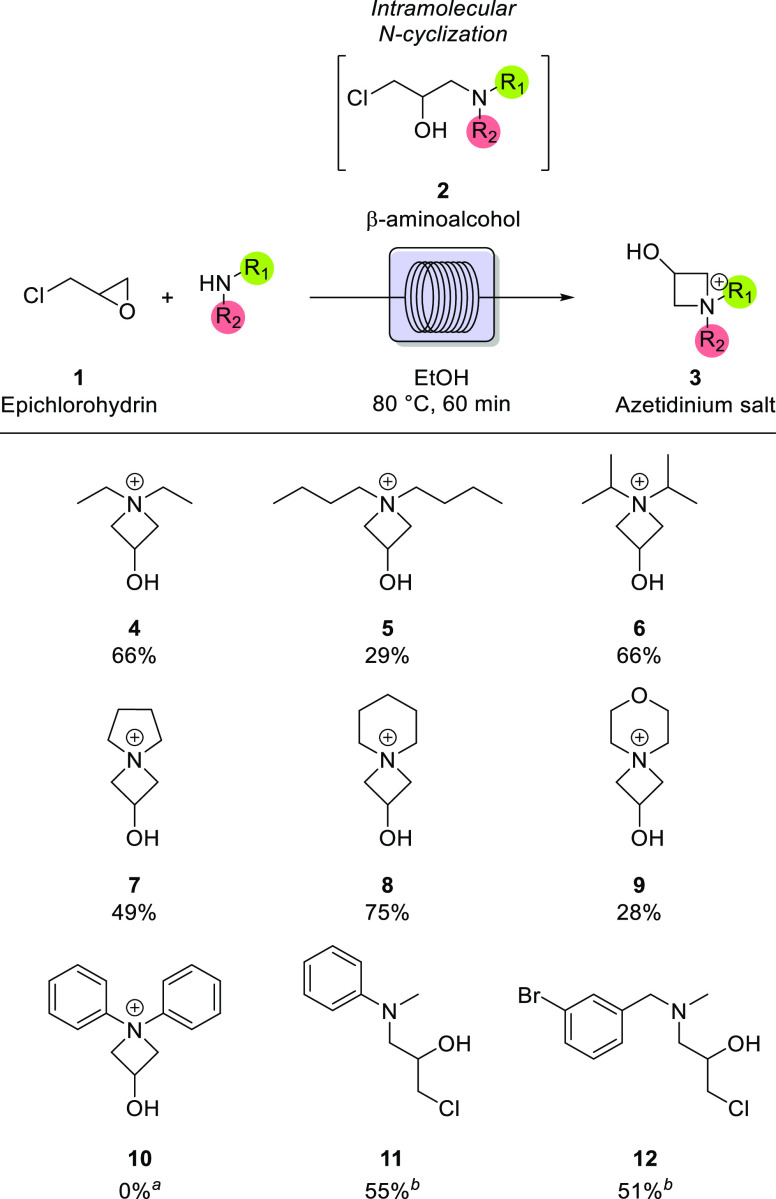
Continuous-flow screening of substrates. ^a^As discussed in the text, the aminolysis is affected by the steric hindrance and negative inductive effect of diphenylamine, resulting in the absence of product formation. ^b^The reaction stops at the formation of the open chain intermediate.

Finally, the influence of the reactivity of secondary amines with respect to azetidinium formation has been evaluated using different substrates (Figure 3). Aliphatic amines appear to be more suitable for azetidinium formation, due to the increased electron availability on the N atom given by the inductive effect of substituents. Product formation with noncyclic amines, like diethylamine **4** and dibutylamine **5** (66% and 29% yields, respectively), is particularly favored, due to the absence of negative inductive effects. Moreover, steric hindrance does not appear to be prominent with alkylic amines, as shown by product **6** (66%). Also, the ring strain of the amine appears to be a critical factor in the reactivity of the system. More constrained amines, like pyrrolidine, are suitable for the formation of the azetidinium **7**, observed in 49% yield, but unfavored if compared to azetidinium **8**, observed in a 75% yield. This kind of reactivity is probably due to the formation of an azaspirocyclic motif positively charged on the N atom, whose instability is a function of ring strain. Even if morpholine is a nonconstrained cyclic amine, the low yield of **9** (28%) highlights a correlation between the basicity of amines and their reactivity: in fact, amines that furnished products from **4** to **8** have a p*K*_a_ of ∼11, whereas morpholine is slightly more acidic (p*K*_a_ ∼ 8). This trend can also be observed in **10–12**, where reactivity is heavily influenced by electronic factors. A negative inductive effect is prominent in the aminolysis mediated by diphenylamine, in which product **10** is not observed (0%); this effect is less accentuated with *N*-methylaniline, in which the positive inductive effect of the alkylic substituent is balanced by the negative one of the phenyl moiety, leading to the formation of open chain intermediate **11** (55% yield). Also using sterically hindered amines, but with favorable electronic effects and appropriate basicity, the formation of the azetidinium product is inhibited, stopping the reaction at the formation of intermediate **12** (51% yield). It has to be remarked that all of the yields reported in Figure 3 refer to the compounds after flow synthesis and concentration in vacuum. In the case of incomplete conversion, the unconverted epichlorohydrin and the amine evaporate during concentration, leading to a clean product.

**Figure 4:**
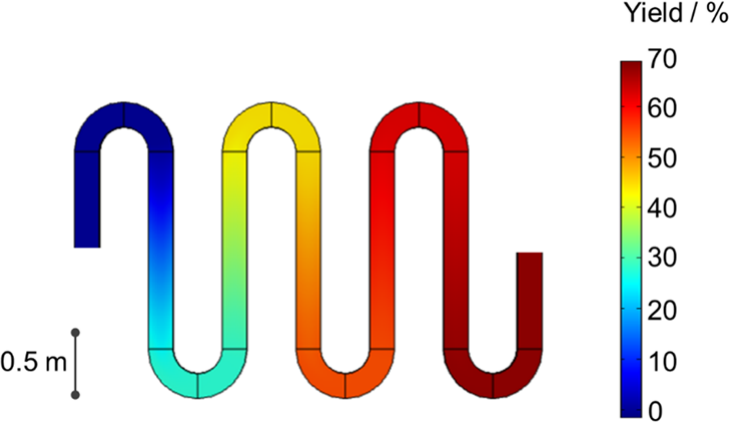
CFD simulation for the continuous-flow synthesis of azetidinium salt from epichlorohydrin with diethylamine, showing the laminar flow rate and good fitting between the modeling and the experimental data.

To study the scalability of our process, all of the reactions have been carried out from 1 to 4 g scale; thus, all yields presented in this work and in Figure 3 can be considered as those for a scale-up process. This points to the efficiency of the method, which can generate azetidiniums quickly, in large amounts, and in very high purity: in fact, in all cases, only the product remains in the crude after rotatory evaporation (as demonstrated also by the NMR spectra in the Supporting Information), pointing to the high selectivity of the route. We have developed a computational fluid dynamic (CFD) model to describe this effect, as well as the fluid patterns and interfacial mass transfer in our flow reactor (Figure 4). The CFD simulation is in good quantitative agreement with the experiments, showing an ∼70% yield of **3** at the outlet of the reactor, in line with the experimentally observed 66% yield. The model indicates that the dominant transport mechanism is a laminar flow pattern, in line with previous literature data.^39^

### 2.4 Mechanistic and Kinetic Studies

**Figure 5:**
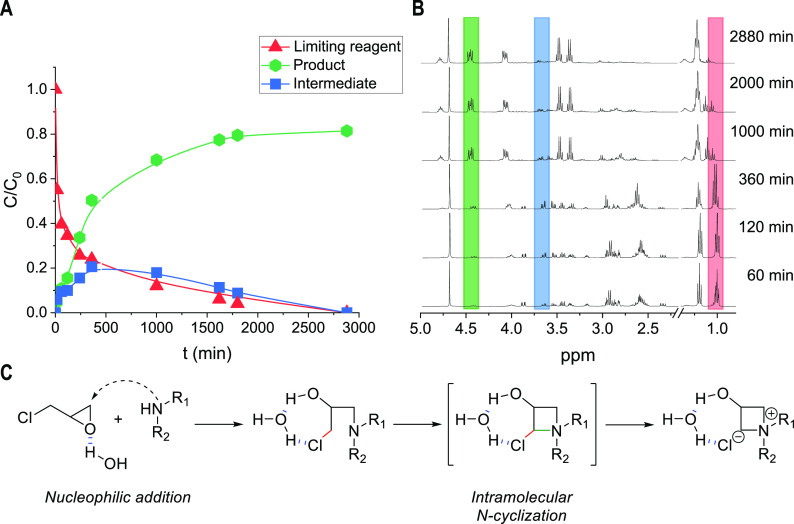
(A) Variation of the concentrations of species during the reaction. (B) NMR analysis *in operando*. (C) Proposed mechanism for the reaction.

We have finally conducted kinetic studies, carrying out the reaction in deuterium oxide as the solvent. This is possible by withdrawing and immediately analyzing the solution at 25 °C. Monitoring the reaction in time, we have observed an increase in the level of product formation, which is initially slower, due to intermediate formation, whose rearrangement to the product makes the azetidinium formation more visible at longer reaction times (Figures 5A). In particular, product selectivity data confirm this hypothesis, as this parameter also decreases with time at higher temperatures; this trend can be justified considering polymerization side reactions. The high conversion data obtained from the beginning of the reaction at all temperatures demonstrate the high reaction rate of intermediate formation; moreover, the low yield and selectivity for the intermediate show the high rate of the N-cyclization reaction, for an overall fast process. This hypothesis is confirmed by following characteristic peaks of the reagents, product, and intermediate in NMR spectra (Figure 5B), revealing a decrease in the intensity in time of the diethylamine peak (t, δ 1.00), contextual to an increase in the height of azetidinium peaks (dd, δ 4.49–4.58) and to an earlier increase and successive decrease in intensity for the intermediate signal (dd, δ 3.64–3.70). The concentrations of species have been calculated by integrating these peaks, and the variation of the concentration with time is shown in Figure 5A. On the basis of these results, we can state that the reaction mechanism in batch and flow mode involves the fast formation of the 1-chloro-*N*,*N*-(diethylamino)propan-2-ol intermediate, which rearranges quickly, giving the final azetidinium product. In the reaction mechanism, an important role is played by the presence of a polar solvent (such as water or EtOH), which activates the epichlorohydrin and initiates the nucleophilic addition, stabilizing both the intermediate and the product as illustrated in Figure 5C. However, for the case of a flow reaction, this mechanistic effect has to be combined with practical aspects, and thus, EtOH has been selected as a solvent to avoid clogging issues in the microreactor and solubilize completely the reagents.

## 3 Conclusions

In summary, we have reported the aminolysis of epichlorohydrin with diethylamine, elucidating the role of solvation, reaction time, and temperature on the reaction yield. The flow method has proved to be highly efficient, giving the corresponding azetidinium salt **3** in higher yields compared to the batch method. In addition to that, we have shown that the continuous process is faster than the batch reaction. Moreover, the intrinsic automation of the flow setup plays a key role in the simplification of the process, avoiding time-consuming and potentially harmful operations, such as adding dropwise epichlorohydrin. The proposed continuous-flow protocol appears to be suitable for a scaled-up synthesis and for different kinds of secondary amines, showing the good flexibility of the procedure.

## 4 Experimental Section

### 4.1 Characterizations

Various amines and epichlorohydrin were purchased from Sigma-Aldrich. ^1^H and ^13^C NMR spectra were recorded on a Bruker 400 MHz spectrometer. ^1^H and ^13^C NMR chemical shifts are reported in parts per million downfield from tetramethylsilane.

### 4.2 Batch Synthesis of 3-Hydroxyazetidinium Chloride

Epichlorohydrin (1 equiv, 28 mmol, 2.6 g, 2.8 M) was added dropwise to a stirred solution of amine (1 equiv, 28 mmol) in the appropriate solvent (5 mL), shown in Table 1, stirring at the desired temperature for 1 h. The crude mixture obtained was concentrated under vacuum and analyzed by NMR, using dibromomethane as an internal standard. No further purification was needed.

### 4.3 Continuous-Flow Synthesis of 3-Hydroxyazetidinium Chloride

A solution of epichlorohydrin (2.8 M in EtOH, flow rate of 0.5 mL min^–1^) and a solution of amine (2.8 M in EtOH, flow rate of 0.5 mL min^–1^) were introduced into the reactor by HPLC pumps, with a residence time of 60 min. The crude mixture obtained is concentrated under vacuum and analyzed by NMR, using dibromomethane as the internal standard. No further purification is needed.

Continuous-flow reactions were conducted in a UNIQSIS FlowLab unit, consisting of two HPLC pumps, a stainless steel T-mixer, a HotCoil heater reactor station, and a back-pressure valve regulator of 10 bar. All of the reactions were carried out in a 60 mL stainless steel coil reactor, with an internal diameter of 1 mm.

#### 4.3.1 1,1-Diethyl-3-hydroxyazetidin-1-ium (**4**)

Colorless oil (66%, 3.0 g); ^1^H NMR (400 MHz, DMSO-*d*_6_) δ 6.65 (d, *J* = 7.0 Hz, 1H), 4.66–4.57 (m, 1H), 4.44 (dd, *J* = 12.0, 7.2 Hz, 2H), 4.12 (dd, *J* = 12.0, 7.2 Hz, 2H), 3.52–3.47 (q, *J* = 7.2 Hz, 2H), 3.38–3.33 (q, *J* = 7.2 Hz, 3H), 1.12 (t, *J* = 7.2 Hz, 6H); ^13^C{^1^H} NMR (101 MHz, DMSO-*d*_6_) δ 70.3, 56.0, 55.8, 53.9, 23.6, 8.3, 7.9; HRMS (ESI-QIT) *m*/*z* calcd for C_7_H_16_NO^+^ [M]^+^ 130.1226, found 130.2485.

#### 4.3.2 1,1-Dibutyl-3-hydroxyazetidin-1-ium (**5**)

Colorless oil (29%, 0.7 g); ^1^H NMR (400 MHz, CDCl_3_) δ 6.85 (s, 1H), 4.89–4.86 (m, 1H), 4.59 (dd, *J* = 11.6, 7.1 Hz, 2H), 4.39 (dd, *J* = 11.6, 7.1 Hz, 2H), 2.52–2.34 (m, 4H), 1.28–1.10 (m, 8H), 0.85 (t, *J* = 7.2 Hz, 6H); ^13^C{^1^H} NMR (101 MHz, CDCl_3_) δ 70.6, 54.1, 29.3, 20.5, 14.0; HRMS (ESI-QIT) *m*/*z* calcd for C_11_H_24_NO^+^ [M]^+^ 186.1852, found 186.2336.

#### 4.3.3 3-Hydroxy-1,1-diisopropylazetidin-1-ium (**6**)

Colorless oil (66%, 2.5 g); ^1^H NMR (400 MHz, D_2_O) δ 4.64 (dd, *J* = 13.5, 6.4 Hz, 1H), 4.35 (dd, *J* = 13.9, 7.5 Hz, 2H), 4.15 (dd, *J* = 13.9, 6.0 Hz, 2H), 3.80 (dtt, *J* = 12.9, 6.5, 3.1 Hz, 2H), 1.38 (t, *J* = 6.6 Hz, 12H); ^13^C{^1^H} NMR (101 MHz, D_2_O) δ 61.14, 60.93, 60.08, 57.43, 16.24; HRMS (ESI-QIT) *m*/*z* calcd for C_9_H_20_NO^+^ [M]^+^ 158.1539, found 158.2225.

#### 4.3.4 2-Hydroxy-4-azaspiro[3.4]octan-4-ium (**7**)

Colorless oil (49%, 3.5 g); ^1^H NMR (400 MHz, DMSO) δ 6.62 (s, 1H), 4.65–4.6 (m, 1H), 4.50 (dd, *J* = 11.6, 6.9 Hz, 2H), 4.24 (dd, *J* = 11.6, 5.7 Hz, 2H), 3.68–3.64 (m, 2H), 3.61–3.55 (m, 2H), 1.97–1.93 (m, 4H); ^13^C{^1^H} NMR (101 MHz, DMSO) δ 71.27, 64.41, 62.98, 21.51, 21.29; HRMS (ESI-QIT) *m*/*z* calcd for C_7_H_14_NO^+^ [M]^+^ 128.1070, found 128.2299.

#### 4.3.5 2-Hydroxy-4-azaspiro[3.5]nonan-4-ium (**8**)

Yellow oil (75%, 3.0 g); ^1^H NMR (400 MHz, DMSO) δ 6.61 (d, *J* = 6.7 Hz, 1H), 4.69–4.57 (m, 1H), 4.50–4.38 (m, 2H), 4.10 (dd, *J* = 11.9, 5.5 Hz, 2H), 3.54 (dd, *J* = 11.2, 5.7 Hz, 3H), 3.39 (dd, *J* = 17.9, 12.2 Hz, 3H), 2.67–2.33 (m, *J* = 45.7, 25.3, 21.0 Hz, 6H), 1.72–1.63 (m, *J* = 9.9, 5.1 Hz, 4H), 1.59–1.51 (m, 3H), 1.46–1.32 (m, 4H); ^13^C{^1^H} NMR (101 MHz, DMSO) δ 70.98, 62.16, 60.40, 58.25, 54.65, 25.19; HRMS (ESI-QIT) *m*/*z* calcd for C_8_H_16_NO^+^ [M]^+^ 142.1226, found 142.2198.

#### 4.3.6 2-Hydroxy-7-oxa-4-azaspiro[3.5]nonan-4-ium (**9**)

Orange oil (28%, 1.4 g); ^1^H NMR (400 MHz, DMSO) δ 6.65 (s, 1H), 4.70–4.63 (m, 1H), 4.58 (dd, *J* = 11.9, 6.6 Hz, 2H), 4.24 (dd, *J* = 11.6, 5.2 Hz, 2H), 3.93–3.85 (m, 2H), 3.79 (dd, *J* = 9.2, 3.6 Hz, 4H), 3.64 (dd, *J* = 9.3, 4.5 Hz, 13H), 3.58 (dd, *J* = 7.7, 3.2 Hz, 8H), 3.54 (t, *J* = 4.8 Hz, 3H), 2.79–2.54 (m, 12H), 2.44 (ddd, *J* = 19.1, 9.9, 4.0 Hz, 13H); ^13^C{^1^H} NMR (101 MHz, DMSO) δ δ 61.85, 60.82, 58.29, 54.24, 53.76; HRMS (ESI-QIT) *m*/*z* calcd for C_7_H_14_NO_2_^+^ [M]^+^ 144.1019, found 144.1926.

#### 4.3.7 1-Chloro-3-[methyl(phenyl)amino]propan-2-ol (**11**)

Dark green oil (55%, 3.0 g); ^1^H NMR (400 MHz, DMSO) δ 7.18 (t, *J* = 7.8 Hz, 1H), 6.74 (d, *J* = 8.1 Hz, 1H), 6.63 (t, *J* = 7.1 Hz, 1H), 3.97–3.91 (m, 1H), 3.65 (dd, *J* = 11.1, 4.5 Hz, 1H), 3.59 (dd, *J* = 11.2, 5.4 Hz, 1H), 3.49 (dd, *J* = 14.9, 5.2 Hz, 1H), 3.29 (dd, *J* = 14.9, 7.1 Hz, 1H); ^13^C{^1^H} NMR (101 MHz, DMSO) δ 149.34, 129.43, 116.32, 112.40, 68.67, 56.19, 48.60; HRMS (ESI-QIT) *m*/*z* calcd for C_10_H_15_ClNO^+^ [M + H^+^]^+^ 200.0837, found 200.1086; HRMS (ESI-QIT) *m*/*z* calcd for C_10_H_14_ClNNaO^+^ [M + Na^+^]^+^ 222.0656, found 222.0811.

#### 4.3.8 1-[(3-Bromobenzyl)(methyl)amino]-3-chloropropan-2-ol (**12**)

Colorless oil (51%, 3.9 g); ^1^H NMR (400 MHz, CDCl_3_) δ 7.39 (s, 1H), 7.37–7.31 (m, 1H), 7.21–7.09 (m, 1H), 3.95–3.89 (m, 1H), 3.62 (d, *J* = 5.5 Hz, 1H), 3.58 (s, 1H), 3.50 (d, *J* = 4.6 Hz, 2H), 2.56–2.47 (m, 3H), 2.22 (s, 3H); ^13^C{^1^H} NMR (101 MHz, CDCl_3_) δ 139.99, 132.02, 130.69, 130.08, 127.71, 122.49, 67.39, 61.88, 60.22, 47.18, 42.20; HRMS (ESI-QIT) *m*/*z* calcd for C_11_H_16_BrClNO [M + H^+^]^+^ 292.0098, found 292.0002.

### 4.4 Kinetic Study

Epichlorohydrin (1 equiv, 28 mmol, 2.6 g, 2.8 M) was added dropwise to a stirred solution of diethylamine (1 equiv, 28 mmol, 2 g) in D_2_O (5 mL), stirring at 25 °C for 48 h. Aliquots of the reaction mixture were withdrawn at the indicated time and immediately analyzed by ^1^H NMR, using ACN as the internal standard.

### 4.5 Reactor Modeling

The reactor simulation was performed using the COMSOL Multiphysics (version 6.3) suite, employing the Chemical Reaction Engineering, the Heat Transfer modules, and the physics-based meshing algorithm with a triangular mesh element size set to extremely fine to discretize the reactor. The kinetic parameters were determined by interpolating the experimental batch and flow data. A sinusoidal tube was constructed in lieu of a coil to reduce the computational complexity, given that the effects of the curvature of the coil on velocity, concentration, and temperature are minimal. The equations were solved numerically using Newton’s method, and convergence was ensured by achieving an absolute error estimate of <5 × 10^–4^.

## Supporting Information Available

^1^H and ^13^C NMR spectra of new compounds (PDF)
