# Supplementary material for: Gram-Scale Domino Synthesis in Batch and Flow Mode of Azetidinium Salts
Source: J Org Chem. 2021 Sep 1;86(20):14113–20. doi: 10.1021/acs.joc.1c01487 (PMC8524416; doi:10.1021/acs.joc.1c01487)

Supporting Information

Gram-Scale Domino Synthesis in Batch and Flow Mode of Azetidinium Salts

*Alessandra Sivo,‡ Vincenzo Ruta,‡ and Gianvito Vilé**

Department of Chemistry, Materials, and Chemical Engineering "Giulio Natta", Politecnico di Milano, Piazza Leonardo da Vinci 32, IT-20133 Milano, Italy.

‡ These authors contributed equally.

* Corresponding author. E-mail: gianvito.vile@polimi.it.

Table of Contents

Table S1. Vis-à-vis comparison between batch and flow data S2

Determination of the activation energy S3

Compounds characterization data S3

Table S1. Vis-à-vis comparison between batch and flow data.

Batch process

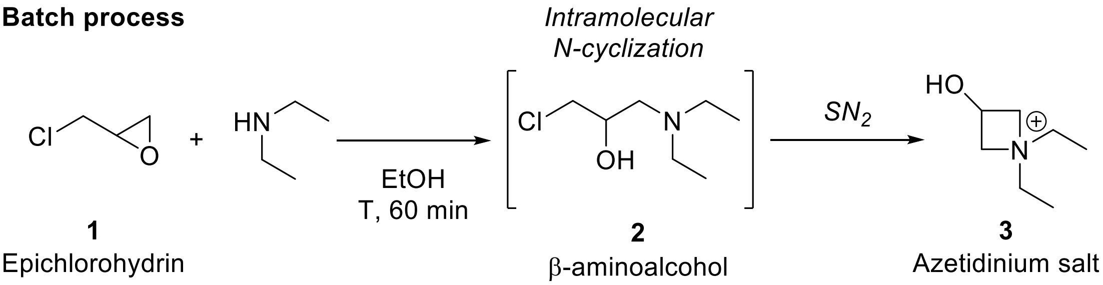

Flow process

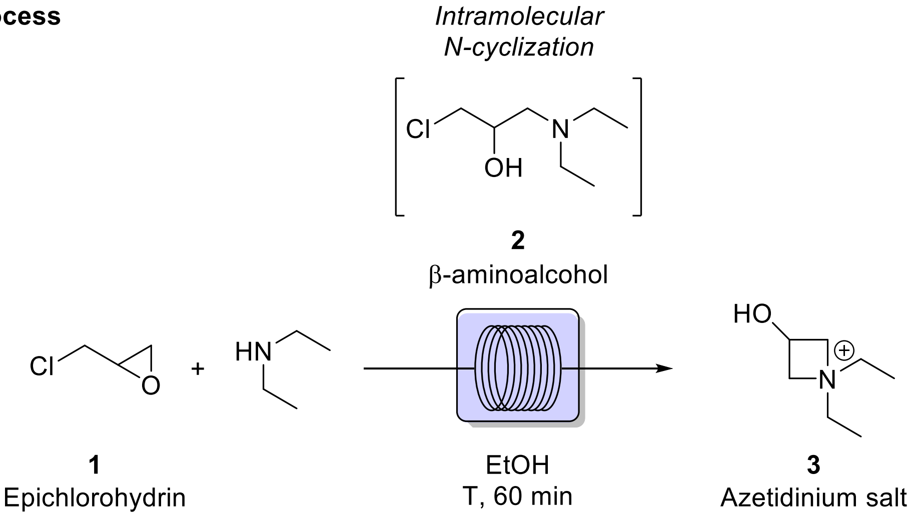

Entry	Solvent	*T* (°C)	*t* (min)	Batch yield (%)[a,b]	Flow yield (%)[a,c]
1	EtOH	25	60	2	0
2	EtOH	60	60	30	63
3	EtOH	80	60	51	66

[a]Calculated by NMR, using dibromomethane as internal standard. [b]Carried out following the 'General procedure for batch synthesis of 3-hydroxyazetidinium chloride' detailed in the experimental section of the manuscript. [c]Carried out following the 'General procedure for the continuous-flow synthesis of 3-hydroxyazetidinium chloride' detailed in the experimental section of the manuscript.

Determination of the activation energy

According to Arrhenius' equation, the activation energy has been calculated plotting the natural logarithm of the azetidinium salt formation as a function of $1/T$. The slope of the obtained line, interpolated in Excel, represents the $-E_a/R$ value, where R is the universal gas constant and E_a is the activation energy.

Compounds characterization data

1,1-diethyl-3-hydroxyazetidin-1-ium (4):

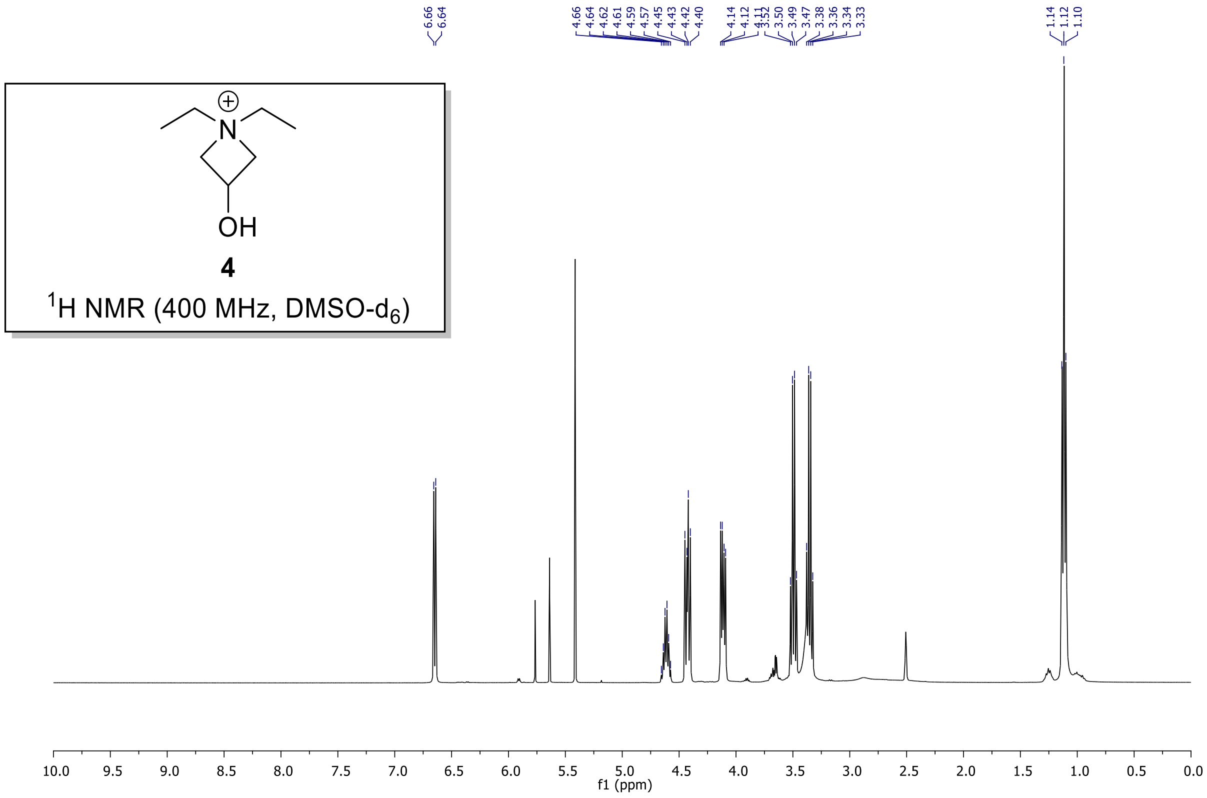

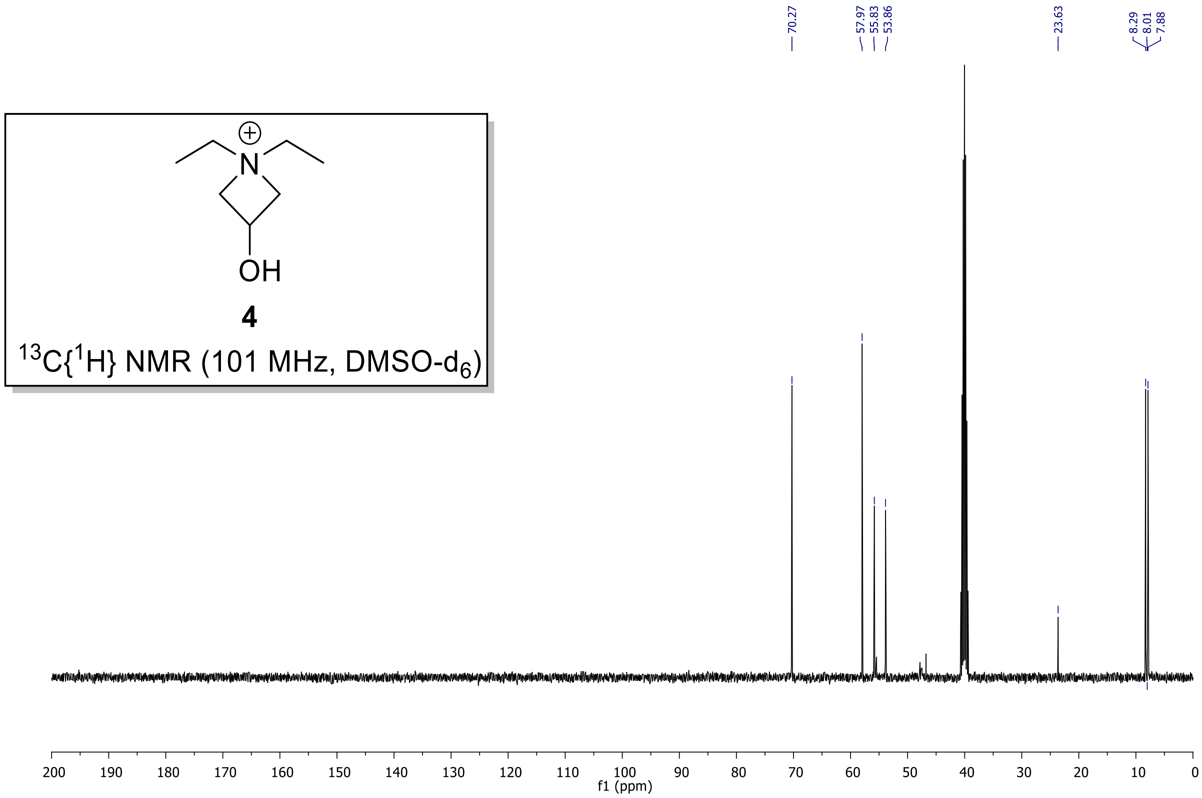
70.27
57.97
55.83
53.86
23.63
8.29
8.01
7.88
OH
4
13C{1H} NMR (101 MHz, DMSO-d6)
200 190 180 170 160 150 140 130 120 110 100 90 80 70 60 50 40 30 20 10 0
f1 (ppm)

1,1-dibutyl-3-hydroxyazetidin-1-ium (5):

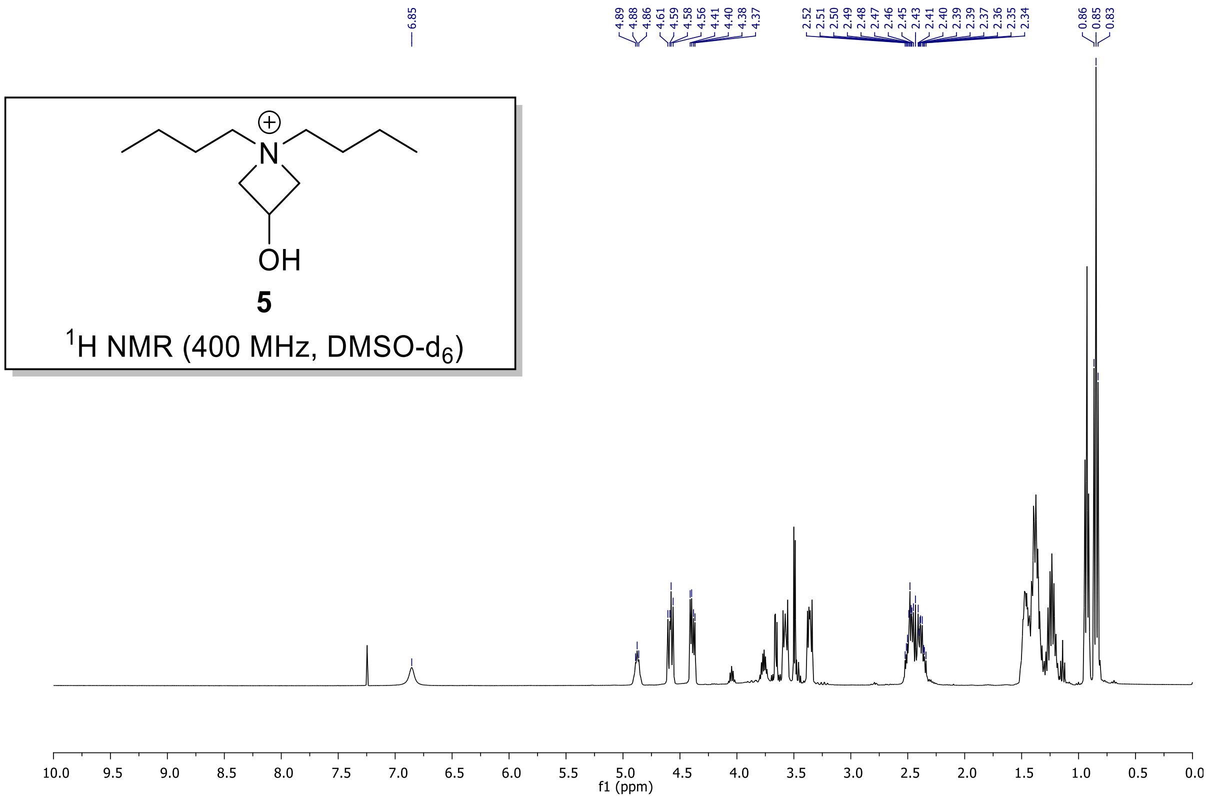

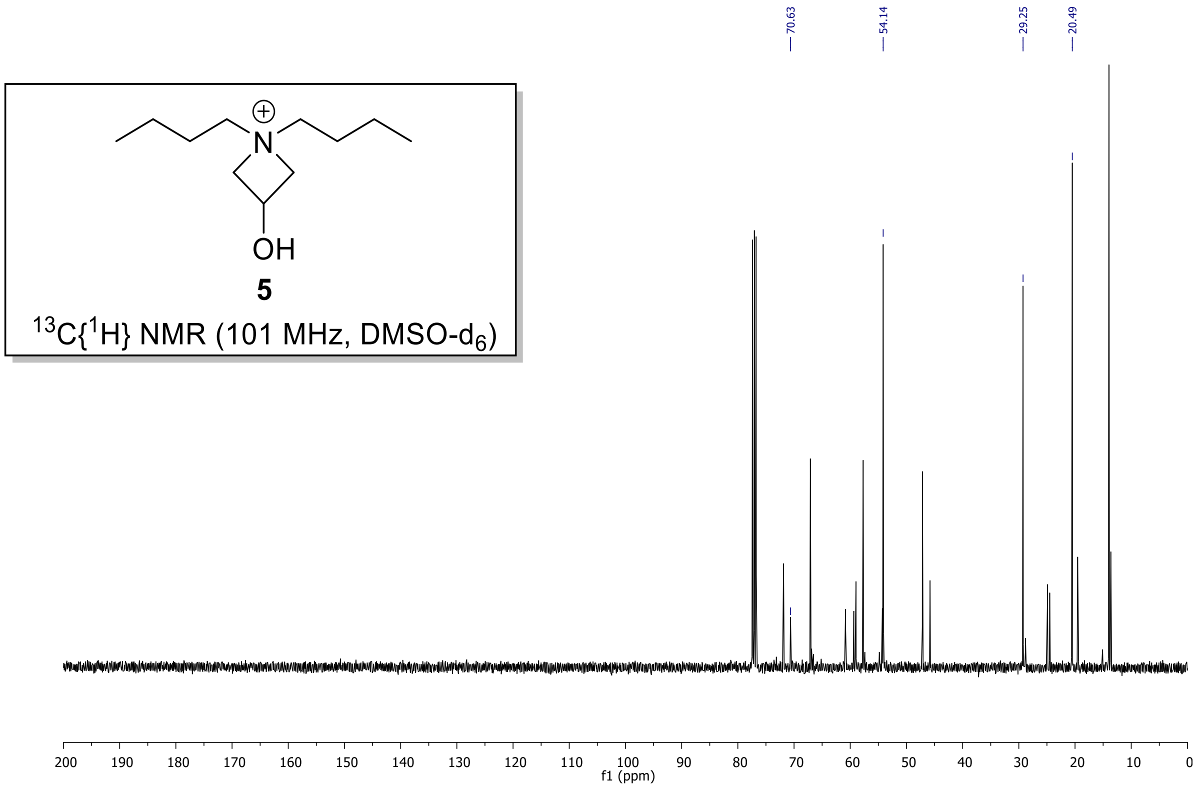

3-hydroxy-1,1-diisopropylazetidin-1-ium (6):

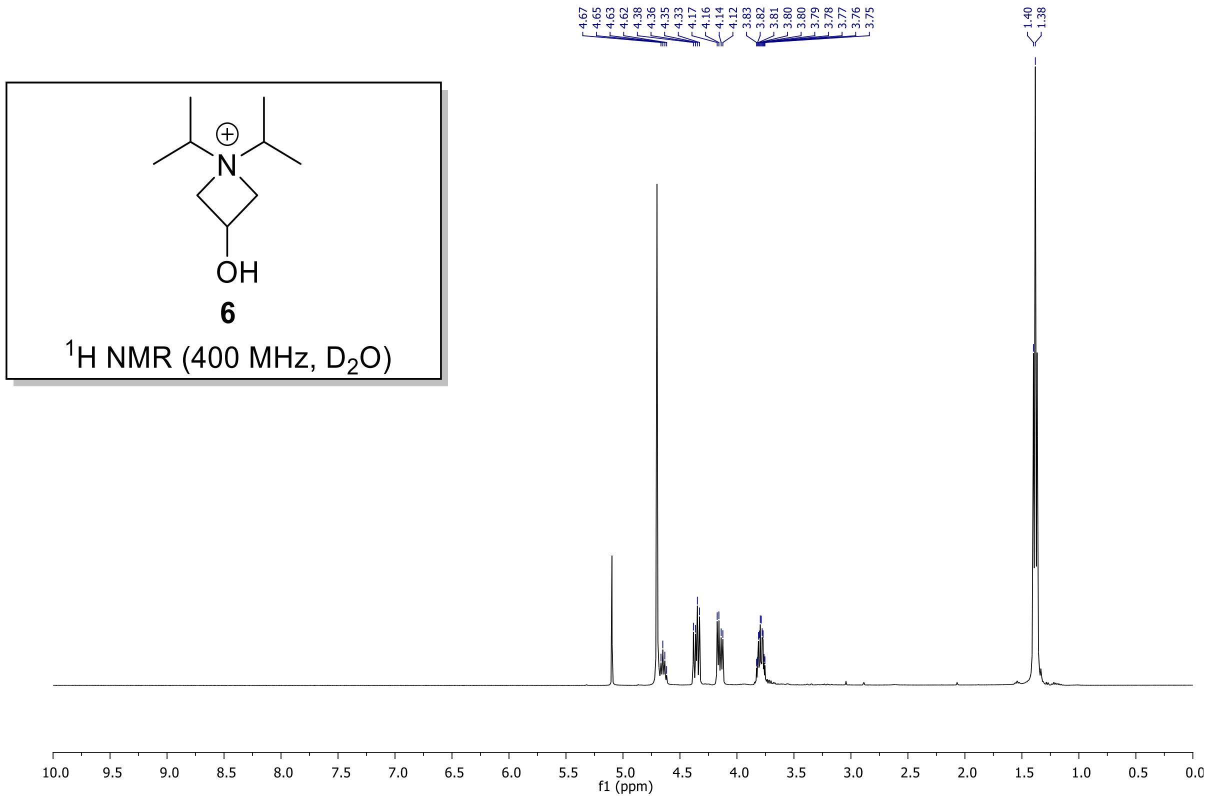

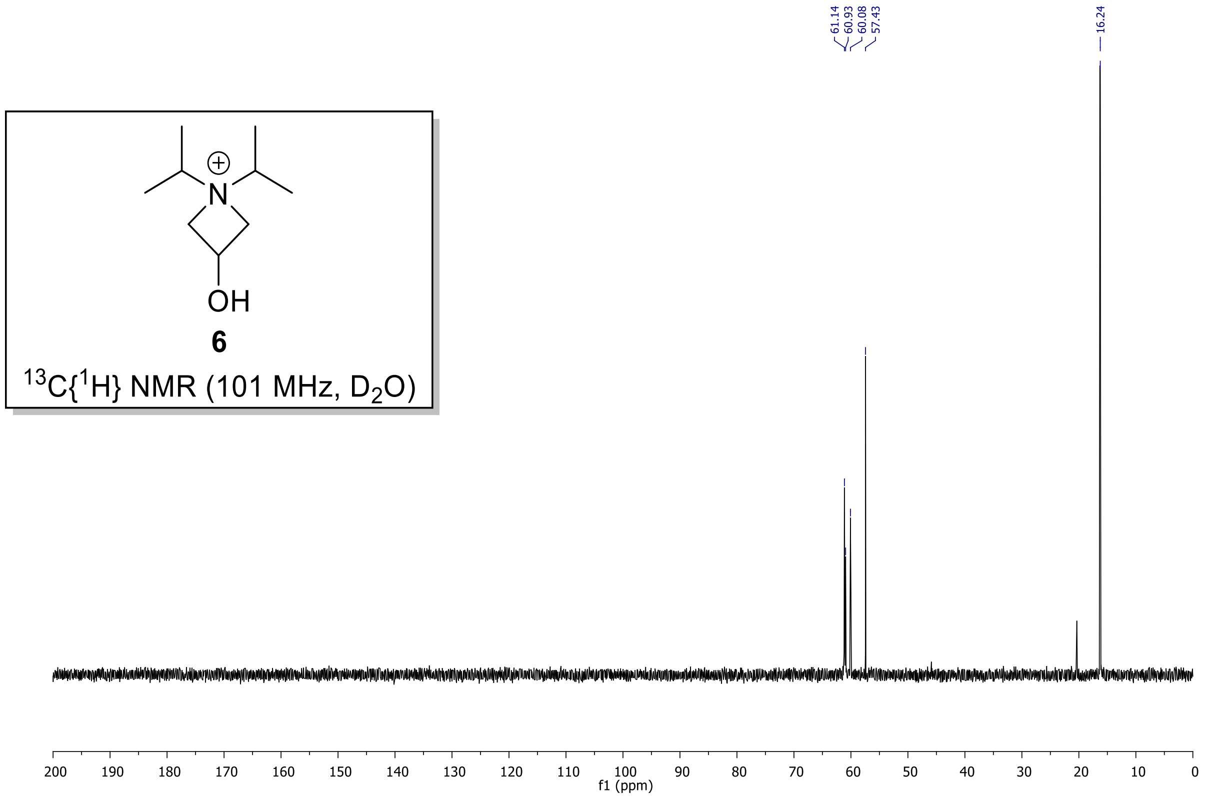

2-hydroxy-4-azaspiro[3.4]octan-4-ium (7):

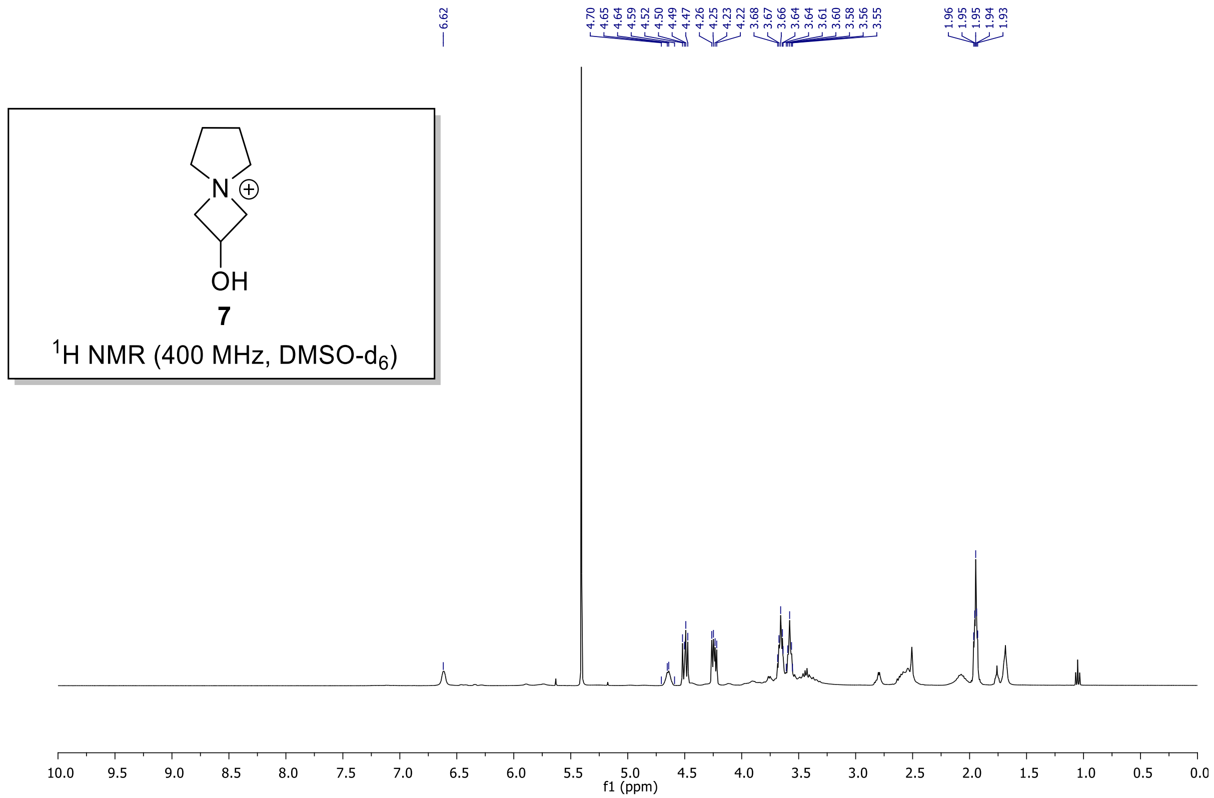

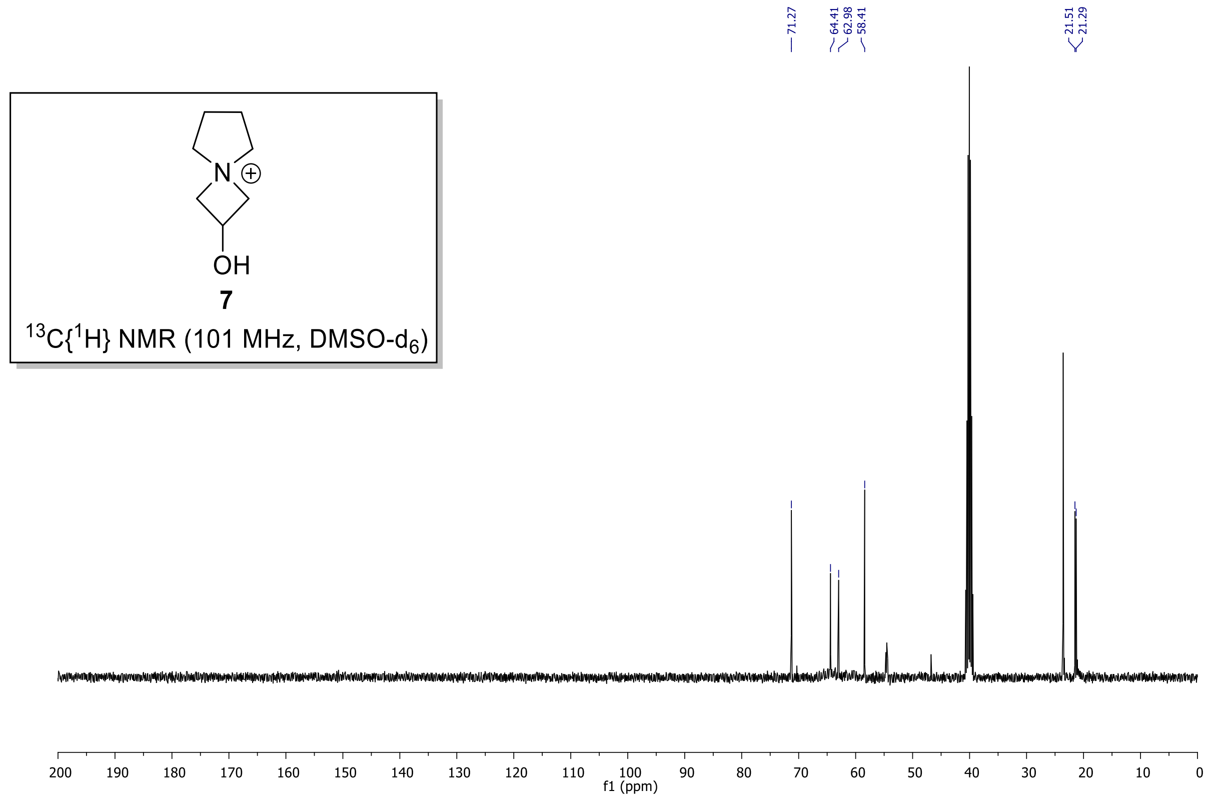

2-hydroxy-4-azaspiro[3.5]nonan-4-ium (8):

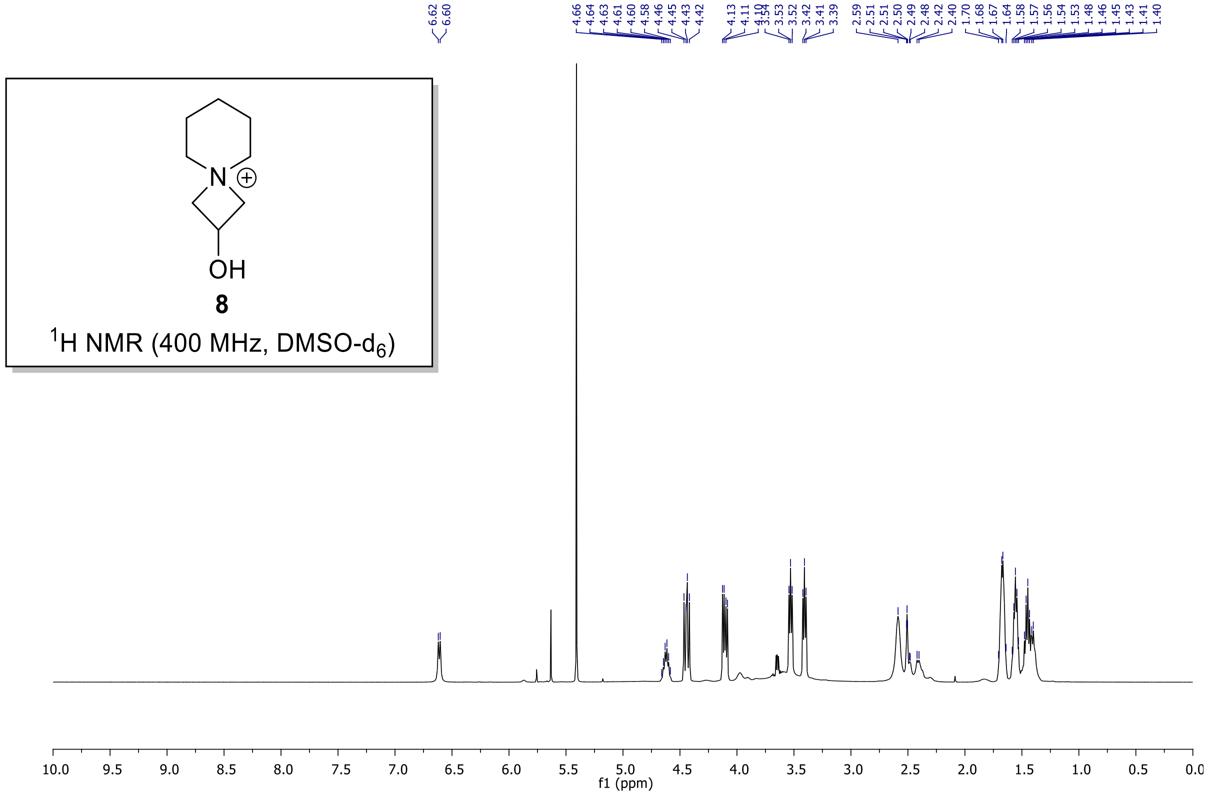

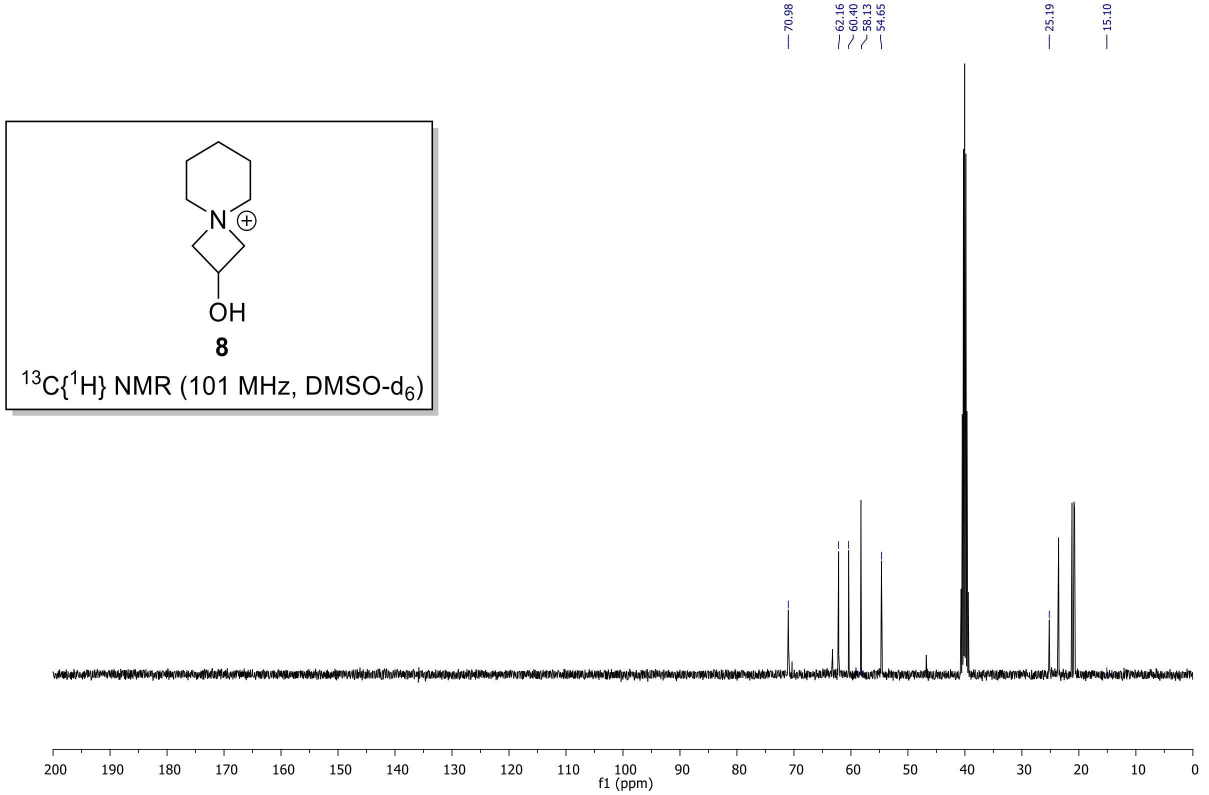

2-hydroxy-7-oxa-4-azaspiro[3.5]nonan-4-ium (9):

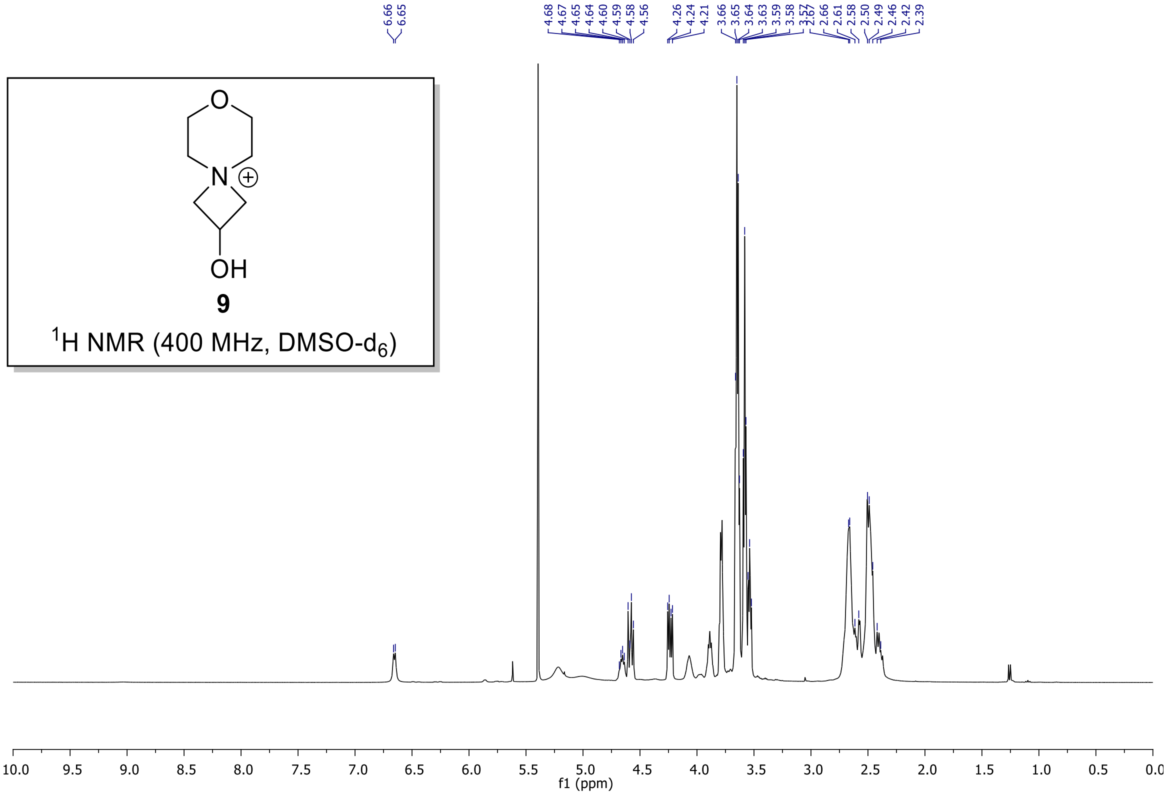

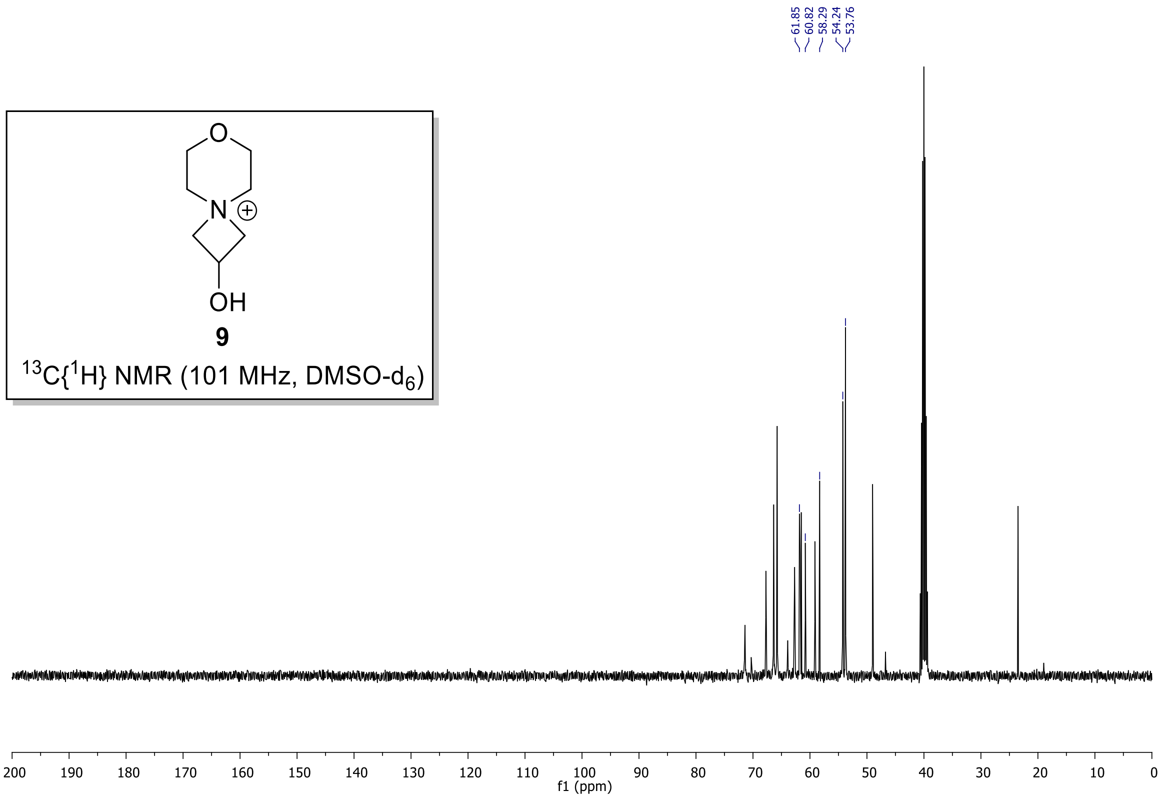

1-chloro-3-(methyl(phenyl)amino)propan-2-ol (11):

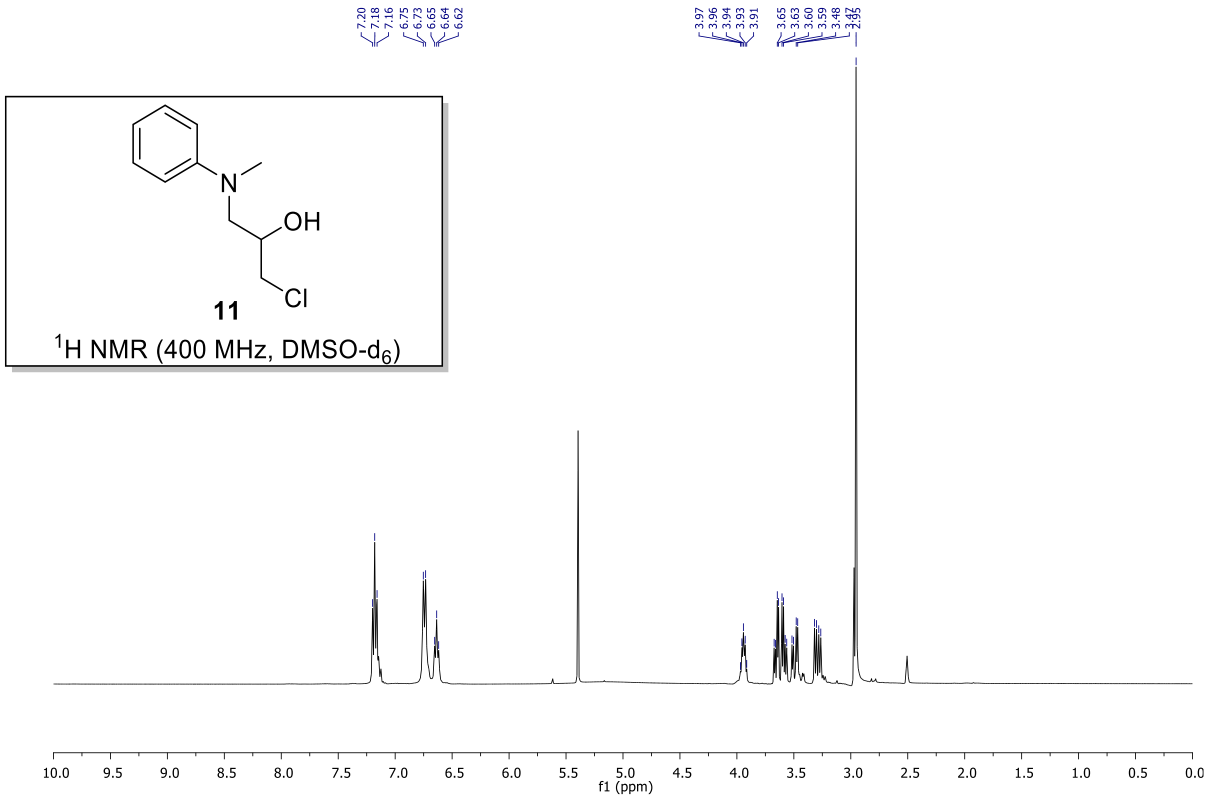

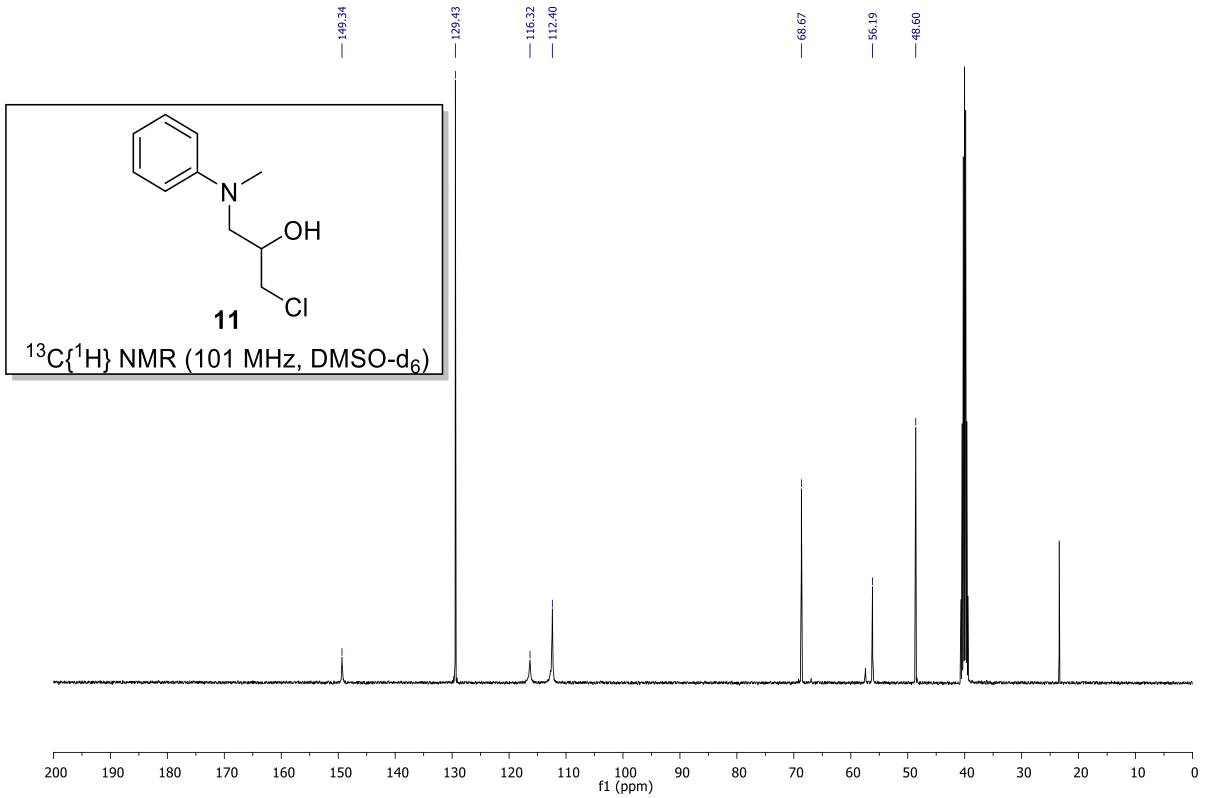

1-((3-bromobenzyl)(methyl)amino)-3-chloropropan-2-ol (12):

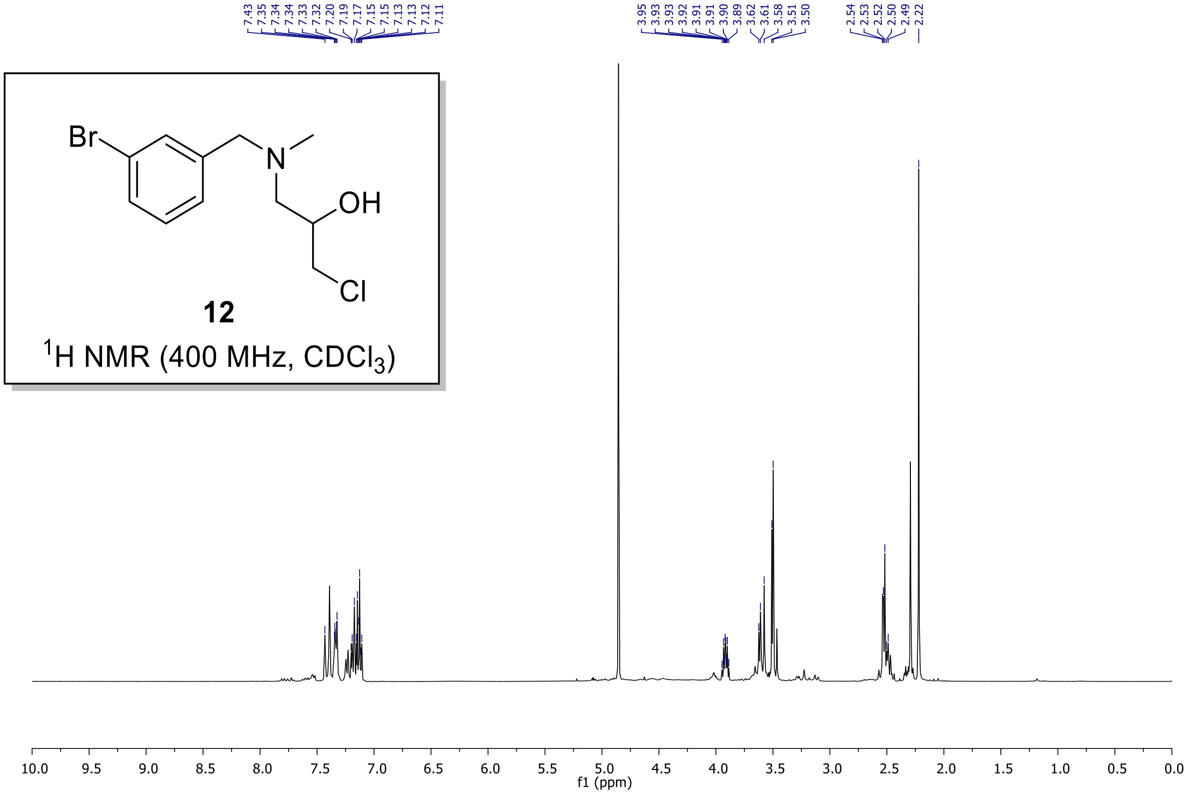

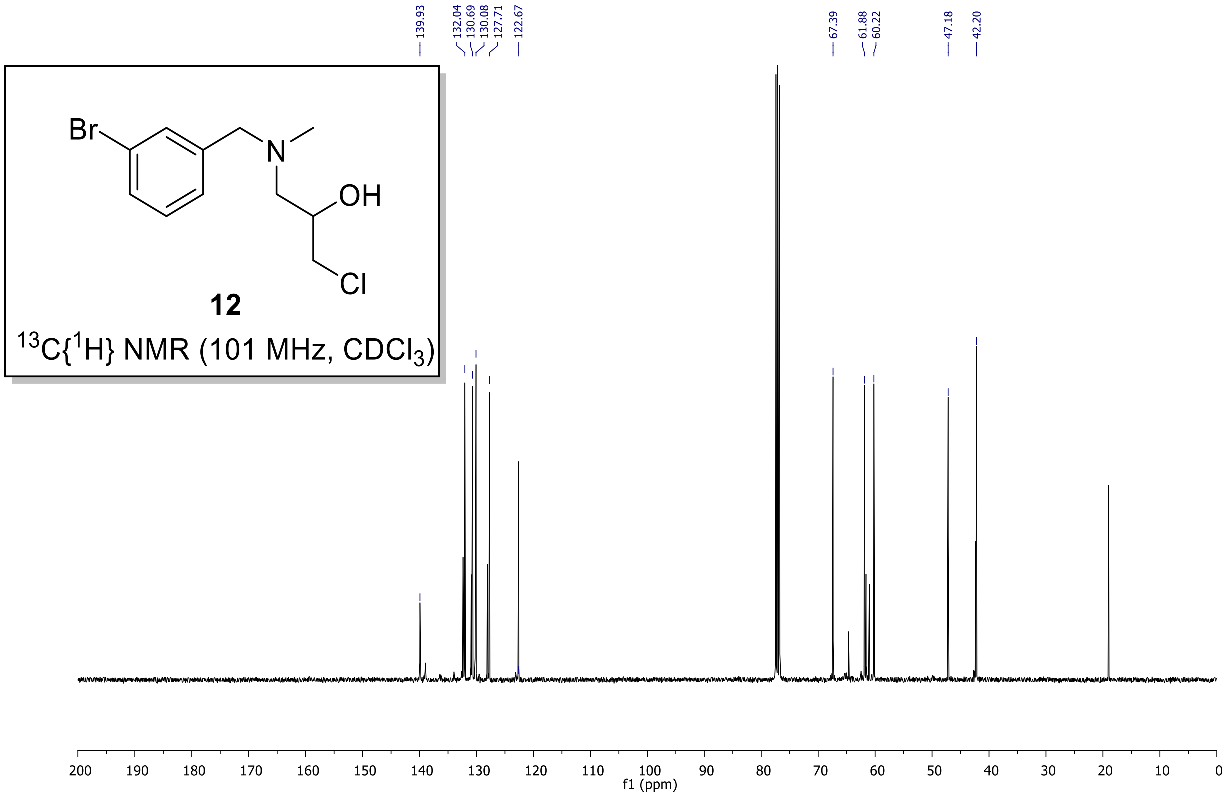

Supplement: Supplementary file 1 — jo1c01487_si_001.pdf [file jo1c01487_si_001.pdf]
